# Comparative analysis of microbiome measurement platforms using latent variable structural equation modeling

**DOI:** 10.1186/1471-2105-14-79

**Published:** 2013-03-05

**Authors:** Xiao Wu, Kathryn Berkow, Daniel N Frank, Ellen Li, Ajay S Gulati, Wei Zhu

**Affiliations:** 1 Department of Applied Mathematics and Statistics, Stony Brook University, Stony Brook, NY, USA; 2 Division of Infectious Diseases, University of Colorado Anschutz Medical Campus, Aurora, CO, USA; 3 Department of Medicine, Stony Brook University, Stony Brook, NY, USA; 4 Department of Medicine, Washington University, St. Louis, MO, USA; 5 Department of Pediatrics, University of North Carolina, Chapel Hill, NC, USA

**Keywords:** Bioinformatics, Latent variable structural equation modeling, Measurement model, Reliability, Repeated measures ANOVA

## Abstract

**Background:**

Culture-independent phylogenetic analysis of 16S ribosomal RNA (rRNA) gene sequences has emerged as an incisive method of profiling bacteria present in a specimen. Currently, multiple techniques are available to enumerate the abundance of bacterial taxa in specimens, including the Sanger sequencing, the ‘next generation’ pyrosequencing, microarrays, quantitative PCR, and the rapidly emerging, third generation sequencing, and fourth generation sequencing methods. An efficient statistical tool is in urgent need for the followings tasks: (1) to compare the agreement between these measurement platforms, (2) to select the most reliable platform(s), and (3) to combine different platforms of complementary strengths, for a unified analysis.

**Results:**

We present the latent variable structural equation modeling (SEM) as a novel statistical application for the comparative analysis of measurement platforms. The latent variable SEM model treats the true (unknown) relative frequency of a given bacterial taxon in a specimen as the latent (unobserved) variable and estimates the reliabilities of, and similarities between, different measurement platforms, and subsequently weighs those measurements optimally for a unified analysis of the microbiome composition. The latent variable SEM contains the repeated measures ANOVA (both the univariate and the multivariate models) as special cases and, as a more general and realistic modeling approach, yields superior goodness-of-fit and more reliable analysis results, as demonstrated by a microbiome study of the human inflammatory bowel diseases.

**Conclusions:**

Given the rapid evolution of modern biotechnologies, the measurement platform comparison, selection and combination tasks are here to stay and to grow – and the latent variable SEM method is readily applicable to any other biological settings, aside from the microbiome study presented here.

## Background

Complex microbial communities, like those of the human gastrointestinal (GI) tract and other environmental specimens, have gained increased attention in recent years, thanks to technological advances in culture-independent methods based on the amplification of 16S rRNA genes [1,2]. The NIH Roadmap Human Microbiome Project (HMP) has undertaken a large scale effort to characterize 16S rRNA sequences from healthy human subjects and from human subjects with various diseases. In the course of conducting the project, the various sequencing centers used both ABI 3730 Sanger sequencing and 454 FLX Titanium pyrosequencing platforms to generate and release reference data from multiple body sites sampled in 300 healthy human subjects [3,4]. Traditional phylogenetic analysis of a sample is performed by amplifying 16S rRNA genes, cloning, and sequencing by the Sanger method [5]. An advantage of this method is the sufficiency of single pass Sanger sequencing of 900–1000 bases for classifying bacteria. Disadvantages include potential cloning bias [6], as well as time and expense, which can be prohibitive for in-depth sampling of complex microbial communities.

Next-generation sequencing (NGS) technology provides a promising alternative to quantifying the microbiome without the limitations of cloning/Sanger sequencing. For instance, a single run of the 454 Life Sciences pyrosequencing platform can produce 1.2 million sequences in 8 hours [7], which would require months or years of work with the older methods. The high throughput per run means the unit cost of NGS is only a fraction of that for Sanger sequencing. The new technology also eliminates the cloning bias by directly sequencing the 16S rRNA genes generated by polymerase chain reaction (PCR). Therefore, high throughput sequencing is ideal if adaptable to meet the requirements needed for microbiome work. However, the main limitation of high throughput sequencing is read length. Reads from NGS technologies are considerably shorter than those from Sanger sequencing. Illumina’s Solexa and Applied Biosystem’s SOLiD platforms generate reads of about 25–100 bases, while 454 sequencing technology reads up to 400–500 bases per sequence. The concern is loss of classification accuracy with shorter sequence reads [8,9]. In addition, the bias associated with PCR amplification is also a concern of PCR based next generation sequencing [10]. Several strategies have been tried to maximize the information obtained from short sequences. One is to target hypervariable regions (HVR) that are most informative for a specific microbiome of interest [11,12]. As a comparison to the Sanger and the NGS methods, quantitative PCR (qPCR) employs primers specific for particular bacterium to detect and quantify bacteria. Although a reliable and accurate quantification measure for the absolute amount of 16S rRNA genes from one specific organism [13], the accuracy of qPCR relies on proper designs of the primers [14].

To date, few attempts have been made to systematically compare and combine different measurement modalities for microbiome analysis. Nossa *et al.*[15] surveyed broad-range 16S rRNA primers for use in 454 pyrosequencing to classify bacteria from the human foregut microbiome. A length of 900 bases long reads were simulated as Sanger sequences and treated as accurate taxonomies. The group concluded that 347 F/803R primers (covering the 16S rRNA V3V4 region) is the most suitable primer pair for pyrosequencing of classification of foregut 16S rRNA genes. Frank *et al.*[16] observed similar results provided by Sanger sequencing and pyrosequencing in the human Nasal Microbiota. One recent work has demonstrated that the measured profile (identification and abundance) of microbial communities depends highly on the selection of sequencing platforms – Sanger sequencing and pyrosequencing with different target regions (V1V3, V4V6, V7V9) yielded varying patterns for different genera [17]. It is thus arduous to compare the accuracies of different sequencing platforms for measuring microbiome compositions in an experimental approach.

Here we propose an alternative analytical approach using the latent variable structural equation modeling (SEM) to compare and integrate microbiome measurements from different measurement platforms. The latent variable SEM treats the true bacterial composition of a sample as the latent (unobserved) variable and estimates the relations between, and the reliabilities of, different measurement platforms, and if necessary, subsequently combines them for a joint analysis with each platform weighed by its reliability [18]. The latent variable SEM includes the repeated measures ANOVA, both the univariate and the multivariate versions, as special cases, and is free from the rigid assumptions of the latter approaches such as weighing each platform equally in the analysis regardless of their reliabilities and assuming equal measurement error variances [19]. Furthermore, as with the repeated measures ANOVA, the latent variable SEM can easily incorporate covariates such as disease phenotypes and genotypes, etc. [20,21] to examine their influences on the underlying microbiome composition/bacteria expression.

In this paper, we demonstrate the latent variable SEM approach through a study of the microbiome in inflammatory bowel diseases (IBD). Our primary goal is to identify the most reliable microbiome measurement platform. A secondary goal is to examine the impact of IBD disease phenotypes (Crohn’s Disease [CD] and ulcerative colitis [UC]) on the enteric microbiota. The measurement platforms compared in this study are: 1) ABI 3730 (Sanger) sequencing of the entire 16S rRNA gene; 2) 454 sequencing of the V1-V3 hypervariable regions; 3) 454 sequencing of the V3-V5 hypervariable region. In the case of a single bacterial taxon, *Faecalibacterium* spp., we compared the three sequencing platforms with an established qPCR assay.

## Methods

In this section, we illustrate the general methodology for platform comparison and combination using latent variable SEM. We start with the simpler latent variable SEM measurement model in which covariates are not involved to better elucidate how latent variable SEM gauges platform reliability and consistency. Subsequently, we introduce latent variable SEM with covariates and describe its two special cases -- repeated measures ANOVA in the univariate and multivariate approaches. To better assist readers with a less mathematical background in this section, each general model is accompanied by the corresponding example from the microbiome study on IBD.

### Measurement model of latent variable SEM

**Figure 1:**
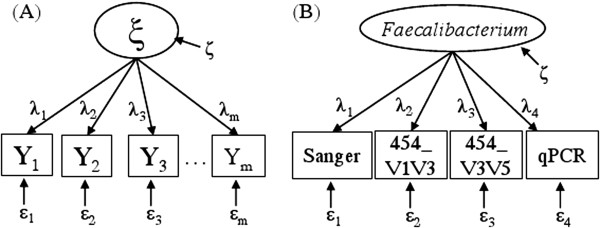
**Path diagram for a latent variable SEM measurement model.** (**A**) The general model with *m* measurements (observed variables) for one latent variable; (**B**) The measurement model with four measurements (Sanger, 454_V1V3, 454_V3V5 and qPCR) for the true (logit-transformed) relative frequency of *Faecalibacterium*.

In latent variable SEM, a latent variable refers to the unknown real value such as the true frequencies of bacteria in the microbiome. The latent variable is linked to its various measurements or indicators through a measurement model. Figure 1(A) describes a measurement model in which the latent variable ξ (for the IBD study, the true frequency of a certain bacteria in a specimen) is gauged through m measurements *Y*_*i*_(*i* = 1, …, *m*) (for the IBD study, measurements from four platforms including Sanger, two 454 windows, and qPCR). Let **Y** = (*Y*_1_, *Y*_2_, ⋯ , *Y*_*m*_)^'^, the latent variable SEM model is a system of linear equations: **Y** = **Λ**ξ + **ε**, where **Λ** = (*λ*_1_, *λ*_2_, ⋯ , *λ*_*m*_)^'^ is the vector of path coefficients showing the expected number of unit changes in the observed variables/measurements for a one-unit change in the true level of ξ. Random errors for the measurements and the latent variable itself are denoted by **ε** = (*ε*_1_, *ε*_2_, ⋯ , *ε*_*m*_)^'^ and ζ respectively. We further assume that all errors are normally distributed and independent with  *Var*(ξ) = $\sigma_{\zeta }^{2}$, *Cov*(*ε*_*i*_, ξ) = 0, *Cov*(*ε*_*i*_, *ε*_*j*_) = 0, and $\mathrm{Var}\left(\varepsilon_{i}\right)=\sigma_{\varepsilon_{i}}^{2}$ (*i*, *j* = 1, …, *m*, *i* ≠ *j*). By convention, **Y** is usually centered about its mean and thus the intercept terms are eliminated.

Let **θ** be the vector of the model parameters including the path coefficients and the error variances and covariances. For the latent SEM model illustrated in Figure 1(A), the population covariance matrix *Σ*(**θ**) of **Y** implied by the SEM model is:

(1) Σθ=EYY'=EΛξ+εξΛ'+ε'=Λσζ2Λ'+covε

Given the multivariate normally distribution of **Y**, one can estimate the model parameters via the traditional maximum likelihood (ML) method that will eventually result in the minimization of the following ML fit function:

(2) $$F_{\mathrm{ML}}=\log\left|\Sigma \left(\theta \right)\right|+\mathrm{tr}\left[S\Sigma^{-1}\left(\theta \right)\right]-\log\left|S\right|-m$$

where S is the sample covariance matrix. This in turn reduces to minimizing the difference between S and *Σ*(**θ**).

To fix ideas, we now illustrate the modeling and estimation of the latent variable SEM in details by setting m = 3 in Figure 1(A). The SEM equations are: *Y*_1_ = *λ*_1_ξ + *ε*_1_, *Y*_2_ = *λ*_2_ξ + *ε*_2_ and *Y*_3_ = *λ*_3_ξ + *ε*_3_, where *E*(*Y*_*i*_) = 0,  *E*(*ε*_*i*_) = 0, $\mathrm{Var}\left(Y_{i}\right)=\sigma_{y_{i}}^{2},$*Var*(ξ) = $\sigma_{\zeta }^{2}$, $\mathrm{Var}\left(\varepsilon_{i}\right)=\sigma_{\varepsilon_{i}}^{2},$*Cov*(*ξ*, *ε*_*i*_) = 0 and *Cov*(*ε*_*i*_, *ε*_*j*_) = 0.

The implied covariance matrix of the model (*its upper triangular portion is omitted in the matrix form due to symmetry) is:

(3) $$\begin{array}{c} \Sigma (\theta )=\Lambda \sigma_{\zeta }^{2}\Lambda '+\mathrm{cov}(\varepsilon )\\ =\left[\begin{array}{ccc} \sigma_{\zeta }^{2}\lambda_{1}^{2}+\sigma_{\varepsilon 1}^{2} & & \\ \sigma_{\zeta }^{2}\lambda_{2}\lambda_{1} & \sigma_{\zeta }^{2}\lambda_{2}^{2}+\sigma_{\varepsilon 2}^{2} & \\ \sigma_{\zeta }^{2}\lambda_{3}\lambda_{1} & \sigma_{\zeta }^{2}\lambda_{3}\lambda_{2} & \sigma_{\zeta }^{2}\lambda_{3}^{2}+\sigma_{\varepsilon 3}^{2} \end{array}\right] \end{array}$$

Following convention for latent variable SEM estimation, we set one of the path coefficients to 1 to assign a scale to the latent variable [22]. This seemingly arbitrary scale assignment has no consequence on the ensuing model estimation because the estimated standardized path coefficients, invariant to this arbitrary scale assignment, will be reported eventually. Thereby without loss of generality, we set *λ*_1_ ≡ 1 in *Σ*(**θ**), and subsequently, by equating *Σ*(**θ**) and S = [Sij], the sample variance covariance matrix, the maximum likelihood estimators of the model parameters soon emerge as:

(4) $${\hat{\lambda }}_{2}=\frac{S_{23}}{S_{13}},{\hat{\lambda }}_{3}=\frac{S_{23}}{S_{12}},{\hat{\sigma }}_{\zeta }^{2}=\frac{S_{12}S_{13}}{S_{23}}$$

(5) $${\hat{\sigma }}_{\varepsilon 1}^{2}=S_{11}-{\hat{\sigma }}_{\zeta }^{2},{\hat{\sigma }}_{\varepsilon 2}^{2}=S_{22}-{\hat{\sigma }}_{\zeta }^{2},{\hat{\sigma }}_{\varepsilon 3}^{2}=S_{33}-{\hat{\sigma }}_{\zeta }^{2}$$

### Platform reliability measure

In order to evaluate the consistency of the measurement platforms, we adopt the concept of reliability originated from the classical test theory by assuming a true score underlies a measure [23]. In the latent SEM measurement model, $R_{y_{i}}^{2}$, the squared correlation coefficient between the latent variable ξ and its measure *Y*_*i*_, is a good reliability measure representing the percentage of variance in a measure that is explained by the latent variable (true score). It is appropriate under very general conditions and, in simple cases, is equal to some of the traditional techniques such as Cronbach’s alpha [22]. For the latent SEM model illustrated in Figure 1(A), ***the reliability measure for the i***^***th***^***platform is*:**

(6) $$R_{y_{i}}^{2}=\rho_{y_{i},\xi }^{2}=\frac{{\mathrm{cov}}^{2}\left(y_{i},\xi \right)}{\mathrm{Var}\left(y_{i}\right)\mathrm{Var}\left(\xi \right)}=1-\frac{\mathrm{Var}\left(\varepsilon_{i}\right)}{\mathrm{Var}\left(y_{i}\right)}$$

The last term in the equation can be interpreted as the proportion of variance in the measure Y_i_ that is explained by the latent variable *ξ* (See Additional file 1 Text S1 for full derivations). The estimated reliability is also closely related to correlations between observed measures. For example, the reliability of y_2_ for the simple case of one latent variable with three measurements (Figure 1A with *m = 3*) is computed as:

(7) $$\begin{array}{l} \;{\hat{R}}_{y_{2}}^{2}=\frac{{\hat{\lambda }}_{2}^{2}{\hat{\sigma }}_{\zeta }^{2}}{{\hat{\sigma }}_{y_{i}}^{2}}={\left(\frac{S_{23}}{S_{13}}\right)}^{2}\times \frac{S_{12}S_{13}}{S_{23}}\times \frac{1}{S_{22}}\\ \;=\frac{S_{12}}{\sqrt{S_{11}S_{22}}}\frac{S_{23}}{\sqrt{S_{22}S_{33}}}\frac{\sqrt{S_{11}S_{33}}}{S_{13}}=\frac{r_{12}r_{23}}{r_{13}} \end{array}$$

Here *r*_*ij*_ is the sample Pearson product moment correlation coefficient between the observed variables *Y*_*i*_ and *Y*_*j*_. Similarly, we have ${\hat{R}}_{y_{1}}^{2}=\frac{r_{12}r_{13}}{r_{23}}\;\mathrm{and}\;{\hat{R}}_{y_{3}}^{2}=\frac{r_{13}r_{23}}{r_{12}}.$ By now we have shown how to compute the R-square from the data, and furthermore, how the R-square is related to the correlations between the observed variables. Suppose the first two of the three measurement platforms are perfectly correlated (*r*_12_ = 1) while the third measure is poorly correlated to the first two with *r*_13_ = *r*_23_ = 0.5. Then we have $R_{y_{1}}^{2}=R_{y_{2}}^{2}=1$, and $R_{y_{3}}^{2}=0.25$. That is, the first two measurements are deemed perfectly reliable on the strength of their perfect consistency, while the third one is considered relatively unreliable due to its poor correlation to the other measures.

The standardized path coefficients are defined as ${\hat{\lambda }}_{i}^{*}={\hat{\lambda }}_{i}\frac{{\hat{\sigma }}_{\zeta }}{{\hat{\sigma }}_{y_{i}}}$. Together with the definition of reliability ${\hat{R}}_{y_{i}}^{2}=\frac{{\hat{\lambda }}_{i}^{2}{\hat{\sigma }}_{\zeta }^{2}}{{\hat{\sigma }}_{y_{i}}^{2}}$, we can easily obtain that ${\hat{R}}_{y_{i}}^{2}=\frac{{\hat{\lambda }}_{i}^{2}{\hat{\sigma }}_{\zeta }^{2}}{{\hat{\sigma }}_{yi}^{2}}={\left({\hat{\lambda }}_{i}^{*}\right)}^{2}$. Therefore, the standardized path coefficient ${\hat{\lambda }}_{i}^{*}$ is indeed the sample correlation between the observed measurement *Y*_*i*_ and the latent variable ζ. The estimated reliability of the ***i***^***th***^ platform is equal to the squared estimated path coefficient in the latent variable SEM measurement model.

### Comparison to repeated measures ANOVA

**Figure 2:**
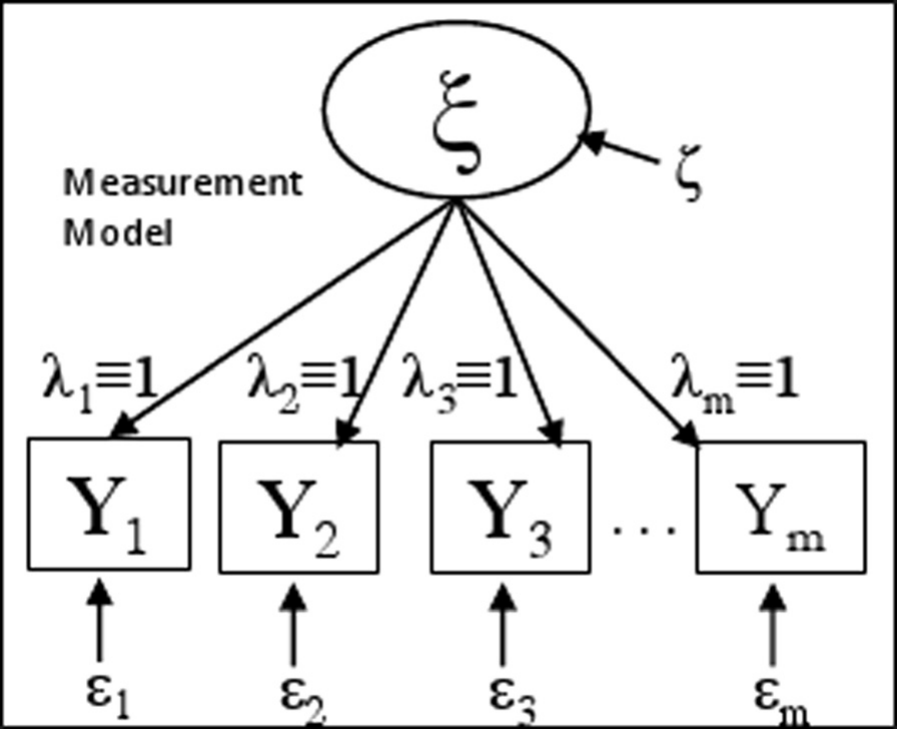
**Path diagram for repeated measures ANOVA.** In comparison to the latent variable SEM model (Figure 1A), repeated measures ANOVA assumes equal path coefficients for both the multivariate and univariate analysis approaches. In addition, for the univariate approach the measurement error variances, Var(ε_i_), are assumed to be equal.

The traditional approach to incorporate multiple repeated measures for the same underlying latent variable is the repeated measures ANOVA. Here we show that the latent variable SEM is a more general model – with the repeated measures ANOVA, both the univariate and the multivariate analysis approaches, as its special cases (Figure 2).

The univariate repeated measures ANOVA model is: **Y** = *Z* + **ε**, where w assume **Y =** (*Y*_1_, *Y*_2_, ⋯ , *Y*_*m*_)^'^ is centered, in analogous to SEM, thus the intercept term is eliminated; Z is the (random) effect of subject; and **ε** = (*ε*_1_, *ε*_2_, ⋯ , *ε*_*m*_)^'^ are independent and identically distributed random errors independent of Z. Therefore **Y** ~ *N*_*m*_(**0**, **Σ**), where omitting the upper triangle of the matrix by symmetry, we have

$$\Sigma =\left[\begin{array}{cccc} \sigma_{z}^{2}+\sigma_{\varepsilon }^{2} & & & \\ \sigma_{z}^{2} & \sigma_{z}^{2}+\sigma_{\varepsilon }^{2} & & \\ \ldots & \ldots & \ldots & \ldots \\ \sigma_{z}^{2} & \sigma_{z}^{2} & \ldots & \sigma_{z}^{2}+\sigma_{\varepsilon }^{2} \end{array}\right].$$

This particular structure of the variance covariance matrix is called “compound symmetry”. The univariate repeated measures ANOVA can be obtained from the more general latent variable SEM shown in Figure 2(A) by imposing equal measurement error variances and equal path coefficients from the measurements to the latent variable. That is, *λ*_*i*_ ≡ 1 and $\sigma_{\varepsilon_{i}}^{2}$ ≡ $\sigma_{\varepsilon }^{2}$ (*i* = 1, 2, … *m*).

The multivariate approach for repeated measures ANOVA allows different measurement error variances but still imposes equal weights to path coefficients from the measurements to the latent variable, that is, *λ*_*i*_ ≡ 1, (*i* = 1, 2, … *m*) as shown in Figure 2. The resulting variance covariance matrix *Σ* for **Y** is:

$$\left[\begin{array}{cccc} \sigma_{z}^{2}+\sigma_{\varepsilon_{1}}^{2} & & & \\ \sigma_{z}^{2} & \sigma_{z}^{2}+\sigma_{\varepsilon_{2}}^{2} & & \\ \ldots & \ldots & \ldots & \ldots \\ \sigma_{z}^{2} & \sigma_{z}^{2} & \ldots & \sigma_{z}^{2}+\sigma_{\varepsilon_{m}}^{2} \end{array}\right].$$

In summary, the repeated measures ANOVA models, both the univariate and the multivariate approaches, are special cases of latent variable SEM with constraints on the error variances and path coefficients. The general latent variable SEM is a more realistic, flexible and better-fitting model to evaluate the latent variable with several measurements, especially when the reliability of each measurement is unclear and the assumption of equal error variances is questionable. This general principle is fully illustrated in the ensuing example of a microbiome study where we compared the latent SEM measurement model with both repeated measures ANOVA models.

### Latent variable SEM with covariates

**Figure 3:**
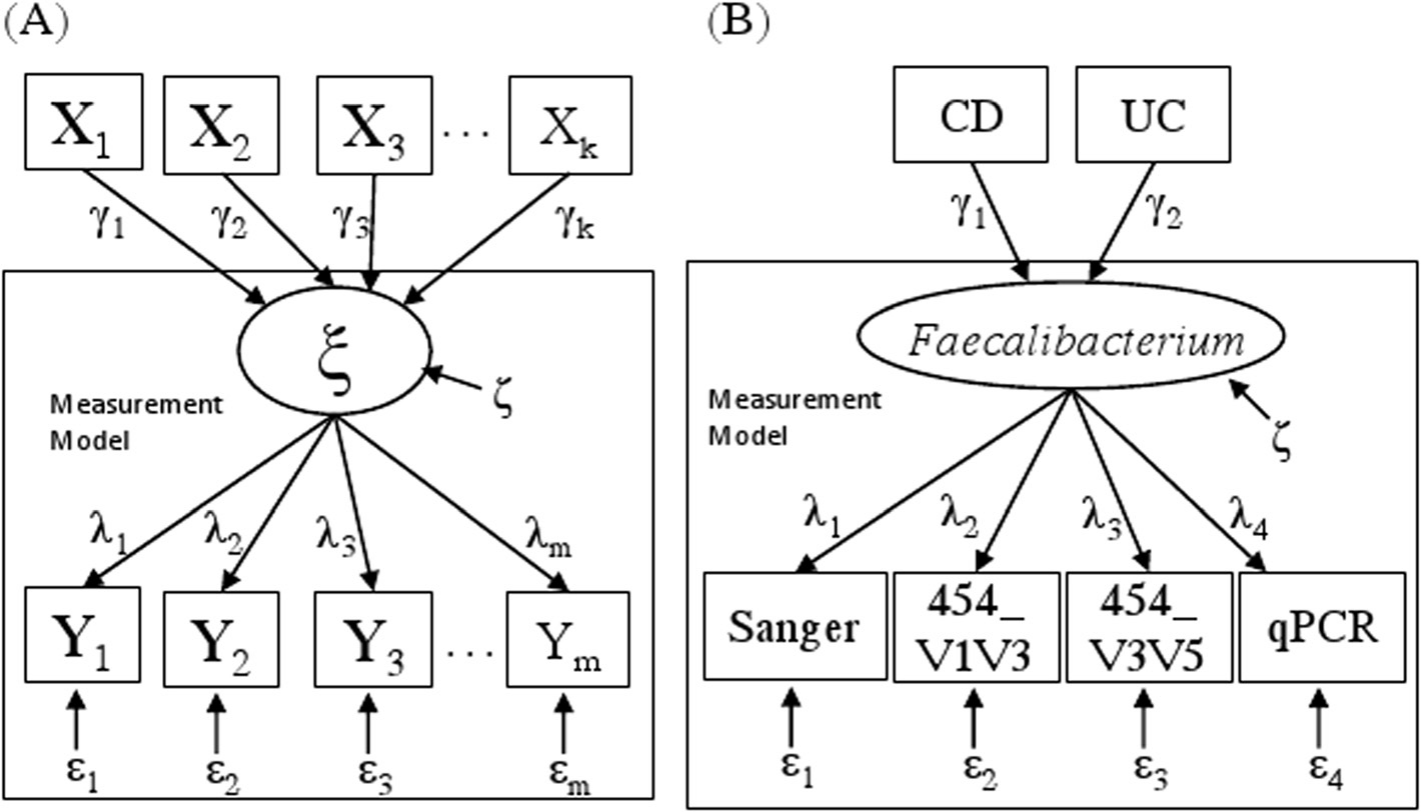
**Path diagram for a latent variable SEM with covariates. **(**A**) A general model with *m* measurements and *k* covariates for one latent variable ξ. (**B**) The model with four measurements (Sanger, 454_V1V3, 454_V3V5 and qPCR) and two covariates -- two binary disease indicators: CD (= 1 for subjects with Crohn’s disease, and 0 otherwise), and UC (= 1 for subjects with ulcerative colitis, and 0 otherwise) for the true/latent (logit-transformed) relative frequency of *Faecalibacterium*.

While one advantage of the latent variable SEM is the ability to simultaneously incorporate multiple measures for the same underlying latent variable in a measurement model as shown in the previous section, SEM also can integrate multiple covariates for a latent variable in the same model. In the ensuing example of IBD, we simultaneously examine the influence of disease phenotypes and genotypes on the underlying bacterial ensemble while incorporating measures from multiple platforms (e.g., Sanger sequencing, 454 pyrosequencing, and qPCR). As illustrated in Figure 3(A), by integrating *k* covariates that might influence the latent variable, the path diagram of the latent variable SEM measurement model illustrated in Figure 1(A) acquires an additional layer.

The SEM model for Figure 3(A) is:

(8) $$\begin{array}{l} Y=\Lambda \xi +\varepsilon \\ \xi =\Gamma 'X+\zeta \end{array}$$

Here, **Y** is a vector of measurement variables for the latent variable *ξ*, and **X** is a vector of independent variables (covariates) affecting the latent variable *ξ*. Both **Y** and **X** have been centered about their means per SEM convention. In addition to the notation in the measurement model, we have **Γ** = (*γ*_1_, *γ*_2_, ⋯ , *γ*_*k*_)^'^ representing the vector of path coefficients from the covariates to the latent variable. The estimation procedure is very similar to the measurement model as well. We can break the covariance matrix *Σ*(**θ**) into a block matrix as follows:

(9) $$\Sigma \left(\theta \right)=\left[\begin{array}{cc} \Lambda \left(\Gamma '\mathrm{cov}\left(X\right)\Gamma +\sigma_{\zeta }^{2}\right)\Lambda '+\mathrm{cov}\left(\varepsilon \right) & \Lambda \Gamma '\mathrm{cov}\left(X\right)\\ \mathrm{cov}\left(X\right)\Gamma \Lambda ' & \mathrm{cov}\left(X\right) \end{array}\right].$$

Thus the parameters can be estimated through minimizing the ML fitting function, or equivalently, by equating **Σ**(**θ**) and S, the sample covariance matrix for both **X** and **Y**.

### Nonparametric analysis of latent variable SEM

In the above, we presented the analysis of latent variable SEM based on the most widely used maximum likelihood estimation (MLE) framework, which depends on normality assumptions. In practice, SEM with continuous variable, including ordinal variables of five categories or more will not have severe problems with non-normality. When the normality assumption is not attainable, one can not directly employ the hypothesis test or confidence interval results. One can employ bootstrap resampling procedures to perform nonparametric significance tests and to construct nonparametric confidence intervals [22,24]. Here we have adopted Efron’s non-parametric bootstrap by re-sampling from the original data with replacement and subsequently obtain the nonparametric bootstrap estimation [25].

In order to fully analyze the following application example on IBD and microbiome, we developed a modified boot.sem function by adapting the boot.sem function from the R package SEM (version 0.9-21) to estimate platform reliability and the standardized latent variable SEM path coefficients and other parameters whenever the normality assumption is not attainable. Our modified boot.sem function is available for free download at http://www.ams.sunysb.edu/~zhu/wei/SEM.html. As an example, the 95% bootstrap confidence intervals of the reliabilities based on the 2.5^th^ and the 97.5^th^ percentiles of the resampled data are shown in the following section.

## Results and discussion

### Data and model descriptions

Inflammatory bowel diseases (IBD), including Crohn’s disease (CD) and ulcerative colitis (UC), are chronic inflammatory conditions of the small intestine and/or the colon. The IBD study reported here includes 39 ileal CD patients, 50 UC patients, and 53 non-IBD control subjects, specimens from which were subjected to microbiome analysis. The abundance of the bacterial genus *Faecalibacterium* (a member of the *Clostridium* Group IV of the phylum Firmicutes) from disease unaffected ileal samples collected from the proximal margin of resected ileum of each subject was determined from four measurement modalities: Sanger sequencing, 454 pyrosequencing of two hypervariable regions of the 16S rRNA gene (V1V3 and V3V5), and quantitative PCR (qPCR) [26]. Assembled Sanger sequences were deposited in GenBank accession HQ739096-HQ821395. 454 V1V3 and V3V5 sequences were deposited in the Sequence Read Archive accession SRX021348-SRX021368, SRX037800-SRX037802. The qPCR assay was performed for *Faecalibacterium prausnitzii* and total bacteria using established primers [27]. *F. prausnitzii* is a predominant species found in the human gastrointestinal microbiome that has been implicated in CD [28,29]. For each sequencing platform, the relative frequency of this bacterial taxon was calculated and then subjected to the empirical logit transformation as described in Li *and others*[26]. The qPCR data (dCT) were converted as qPCR = logit(2^dCT^) so that all four measurements were subjected to the same transformation. The IBD phenotypes (CD and UC) are incorporated as two covariates into the SEM model for an association analysis as well. Path diagrams for the latent variable SEM measurement, and covariate models for *Faecalibacterium* are shown in Figure 1(B) and Figure 3(B) respectively.

### Consistency and reliability of different measurement modalities

Table 1 shows the Pearson correlation among the four measurement modalities for the logit transformed relative frequency of *Faecalibacterium*. The V3V5 pyrosequencing window is the best correlated among all modalities. In contrast, the qPCR data have relatively low correlations with all three sequencing measures, suggesting that the target of qPCR, *F. prausnitzii*, might not represent the full faecalibacterial diversity in the sample set. Therefore, although qPCR is often treated as the gold standard for the quantification of nucleotide sequences, it may be limited by its high dependency on the accurate specification of primers of targets.

**Table 1:** **Pearson correlations among four different measurement modalities for the logit transformed relative frequency of *Faecalibacterium *(N = 142)**

	**Sanger**	**454_V1V3 (*p *value)**	**454_V3V5 (*p *value)**	**qPCR (*p *value)**
Sanger	1	0.828 (<.001)	0.866 (<.001)	0.642 (<.001)
454_V1V3		1	0.887 (<.001)	0.624 (<.001)
454_V3V5			1	0.610 (<.001)
qPCR				1

The reliabilities of these measurement modalities, as estimated by the squared correlation coefficients between measurements and the latent variable, are shown in the Table 2. Again, the V3V5 pyrosequencing window was found to be the most reliable with a reliability score of 0.912, and a correlation of 0.955 to the true underlying *Faecalibacterium* expression.

**Table 2:** **Reliability of each measurement platform in the four-modality latent variable SEM measurement model, and its correlation to the latent variable (true relative frequency of *Faecalibacterium*)**

	**Four- modality measurement model**			
	**Sanger**	**454_V1V3**	**454_V3V5**	**qPCR**
Reliability	0.819	0.857	0.912	0.441
(95% CI)	(0.689, 0.907)	(0.774, 0.917)	(0.865, 0.963)	(0.303, 0.553)
Correlation to the latent variable	0.905	0.926	0.955	0.664
(95% CI)	(0.830, 0.952)	(0.880, 0.958)	(0.930, 0.981)	(0.550, 0.744)

The 95 % confidence intervals are obtained using bootstrap resampling with 100 replications [24].

Because the reliability measure calculated in this model is closely related to the correlations among measurement modalities, and because the two 454 pyrosequencing windows feature the highest correlation (r = 0.887), we also evaluated a three-modality measurement model that dropped the 454 V1V3 data (the less reliable pyrosequencing window). In this independent platform comparison, Sanger sequencing emerged as the most reliable platform among the three modalities with an estimated reliability of 0.911 and an estimated correlation of 0.955 with the underlying *Faecalibacterium* frequency (Table 3, upper half). Result is similar, with Sanger sequencing being the most reliable measurement, if an alternative three-modality comparison was evaluated among Sanger, 454_V1V3 and qPCR (Table 3, lower half).

**Table 3:** **Reliability of each measurement platform in the three-modality latent variable SEM measurement model, and its correlation to the latent variable (true relative frequency of *Faecalibacterium)***

	**Three- modality measurement model**		
	**Sanger**	**454_V3V5**	**qPCR**
Reliability	0.911	0.822	0.452
(95% CI)	(0.775, 1.000)	(0.720, 0.912)	(0.323, 0.610)
Correlation to the latent variable	0.955	0.907	0.672
(95% CI)	(0.880, 1.000)	(0.849, 0.955)	(0.568, 0.781)
	Sanger	454_**V1V3**	qPCR
Reliability	0.851	0.806	0.483
(95% CI)	(0.671, 1.000)	(0.645, 0.905)	(0.350, 0.648)
Correlation to the latent variable	0.922	0.898	0.696
(95% CI)	(0.819, 1.000)	(0.803, 0.951)	(0.592, 0.805)

The 95% confidence intervals are obtained using bootstrap resampling with 100 replications. Two 3-modality models are shown with Sanger, qPCR, and 454_V3V5 in the first model, and 454_V1V3 in the second model.

**Figure 4:**
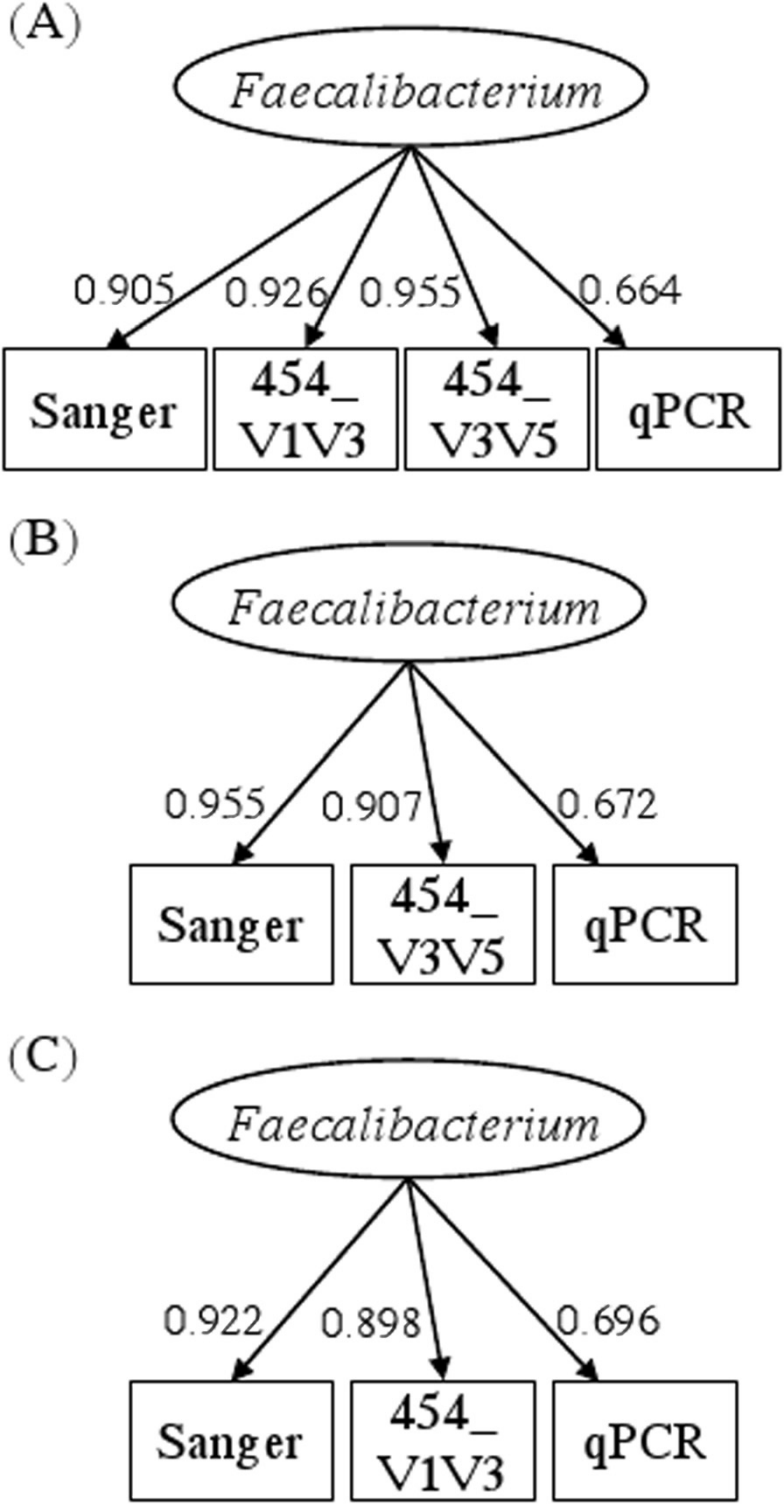
The estimated (A) four-modality (B) three-modality (Sanger, 454_V3V5, qPCR) and (C) three-modality (Sanger, 454_V1V3, qPCR) latent variable SEM measurement models for a study of the inflammatory bowel diseases.

Path diagrams for the measurement models with the estimated standardized path coefficients are shown in Figure 4. As demonstrated above, the standardized path coefficients are indeed the correlations between each measurement and the latent variable.

In addition to *Faecalibacterium*, similar analyses have been performed for several other bacterial taxa which are potentially associated with IBD (with the latent variable SEM results shown in Table 4 below and the corresponding Pearson correlations between the observed variables listed in Additional file 1: Table S1). With the three measurement platforms (Sanger, 454_V1V3 and 454_V3V5) available for these bacterial groups (the qPCR was not performed for these groups, unfortunately), the 454_V3V5 window is shown to be a better measurement platform for *Proteobacteria*, *Actinobacteria*, *Bacteroidetes* and *Firmicutes/Bacilli*, while the 454_V1V3 window is found to be more reliable for *Firmicutes/Clostridia/Clostridiales/ LachnoIV*. This observation is consistent with, and thus further confirmed the point made by the joint panel of human microbiome project in that different 454 pyrosequencing windows may be optimal for different bacterial taxa [4].

**Table 4:** Reliability for more bacterial taxa in the three-modality latent variable SEM measurement model (Sanger, 454_V1V3 and 454_V3V5), and its correlation to the latent variable

	**Three-measurement modality model**		
	**Sanger**	**454_V1V3**	**454_V3V5**
(A) *Proteobacteria*			
Reliability	0.657	0.641	**0.974**
(95% CI)	(0.524, 0.793)	(0.529, 0.724)	**(0.878, 1.000)**
Correlation to the latent variable	0.811	0.801	**0.987**
(95% CI)	(0.724, 0.891)	(0.727, 0.851)	**(0.937, 1.000)**
(B) Firmicutes/Clostridia/Clostridiales/LachnoIV			
Reliability	0.685	**0.923**	0.793
(95% CI)	(0.582, 0.804)	**(0.837, 1.000)**	(0.688, 0.903)
Correlation to the latent variable	0.827	**0.961**	0.890
(95% CI)	(0.763, 0.897)	**(0.915, 1.000)**	(0.829, 0.950)
(C) *Actinobacteria*			
Reliability	0.582	0.854	**0.882**
(95% CI)	(0.424, 0.700)	(0.743, 0.942)	**(0.765, 0.976)**
Correlation to the latent variable	0.763	0.924	**0.939**
(95% CI)	(0.652, 0.837)	(0.862, 0.970)	**(0.875, 0.988)**
(D) *Bacteroidetes*			
Reliability	0.684	0.828	**0.980**
(95% CI)	(0.323, 0.922)	(0.652, 1.000)	**(0.941, 1.000)**
Correlation to the latent variable	0.827	0.910	**0.990**
(95% CI)	(0.569, 0.960)	(0.808, 1.000)	**(0.970, 1.000)**
(E) *Firmicutes/Bacilli*			
Reliability	0.698	0.953	**0.959**
(95% CI)	(0.553, 0.797)	(0.888, 1.000)	**(0.913, 0.995)**
Correlation to the latent variable	0.835	0.976	**0.979**
(95% CI)	(0.744, 0.893)	(0.942, 1.000)	**(0.956, 0.998)**

The 95% confidence intervals are obtained using bootstrap resampling with 100 replications.

### Comparison to repeated measures ANOVA

The model goodness-of-fit indices for the four-modality latent variable SEM measurement models for *Faecalibacterium* are listed in Table 5, and compared to those for the repeated measures ANOVA in both the univariate and the multivariate analysis approaches. SEM relies on several statistical tests to determine the adequacy of model fit to the data. The chi-square test indicates the amount of difference between the expected and the observed covariance matrices (values near zero indicate little difference between expected and observed covariance matrices). The root mean square error of approximation (RMSEA), which is related to the residuals in the SEM model, ranges from 0 to 1 with a smaller RMSEA value indicating better model fit. Acceptable model fit is indicated by an RMSEA value of 0.06 or less [30]. The Comparative Fit Index (CFI) is equal to the discrepancy function adjusted for the sample size. That is, CFI = 1 – d_(proposed model)_/d_(null model)_, where d is equal to the corresponding chi-square minus the degrees of freedom of the model. The CFI ranges from 0 to 1 with a larger value indicating better model fit. Acceptable model fit is indicated by a CFI value of 0.90 or greater [30]. As shown in Table 5, the latent variable SEM (model A) has significantly better Chi-square goodness-of fit index (χ^2^ = 5.089, *p* = 0.079) than model B and C representing the repeated measures ANOVA in the multivariate and univariate approaches respectively. Model A also has relatively better RMSEA index than model B and C. For the CFI criterion, only model A provides good fit with a values above 0.9. ***In summary, the (general) latent variable SEM is the only model that fits the data well as neither of the repeated measures ANOVA models is satisfactory.***

**Table 5:** **Model goodness-of-fit comparison between latent variable SEM and repeated measures ANOVA approach of *Faecalibacterium *based on four measurements (Sanger, 454 pyrosequencing V1V3, 454 pyrosequencing V3V5 and qPCR)**

**MODEL**	**MODEL CONSTRAINT**	**GOODNESS-OF-FIT**	
**A: Latent variable SEM**	set only λ_1_ = 1	Chi-square	5.089 (df = 2) Pr > χ^2^: 0.079
		RMSEA	0.105
		CFI	0.994
**B: Equivalent to repeated measures ANOVA (multivariate approach)**	set all indicator path coefficient λ_i_ ≡ 1 (i = 1, 2, 3, 4)	Chi-square	129.955 (df = 5) Pr > χ^2^: < .001
		RMSEA	0.421
		CFI	0.750
**C: Equivalent to repeated measures ANOVA (univariate approach)**	set all indicator path coefficient λ_i_ ≡ 1; set all indicator error variances to be equal, var (ε_i_) ≡ *σ*^2^ (i = 1, 2, 3, 4)	Chi-square	172.068 (df = 8) Pr > χ^2^: < .001
		RMSEA	0.381
		CFI	0.671

### Estimation of the latent variable SEM model with IBD phenotypes

**Figure 5:**
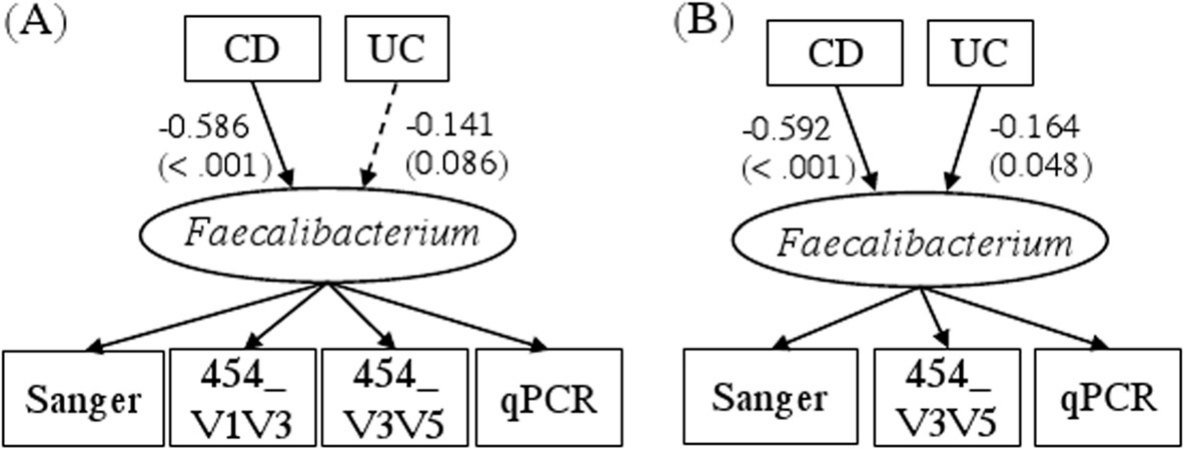
**The estimated (A) four- and (B) three-modality latent variable SEM models examining the effect of two covariates: CD and UC phenotypes with their path coefficients and the corresponding *p*-values (in parentheses).**

In this section, we examine the impact of two IBD phenotypes, Crohn’s Disease (CD) and ulcerative colitis (UC), on the relative frequency of *Faecalibacterium* via latent variable SEM, simultaneously utilizing measurements of the given genus from either all four modalities, or only three distinct modalities (minus the V1V3 window of the 454 pyrosequencing). CD patients are found to have significantly lower relative abundance of *Faecalibacterium* (*p* < .001) in both four- and three-modality latent variable SEM analysis. While UC patients were confirmed to have significant lower average concentration of *Faecalibacterium* in the three-modality model with *p* = 0.048 but only a trend of reduction in the four-modality model (*p* = 0.086) (Figure 5). The difference may lie in the decrease of model parameters for the three-modality model that renders it more powerful to detect the underlying difference than the four-modality model. In accordance to previous reports that low relative frequency of *F. prausnitzii* has been found in ileal CD patients and it has been associated with an increased risk of ileocolonoscopic recurrence of ileal CD [31].

The estimated values of path coefficients in the association study with IBD phenotype are interpreted as follows. Take the three- modality covariate latent variable SEM for example (Figure 5). The relation between the estimated logit transformed true relative frequency (*π*) of *Faecalibacterium* (the latent variable *ξ*) and the phenotypes CD and UC is interpreted as follows:

$$\hat{\xi }=\log\left(\frac{\hat{\pi }}{1-\hat{\pi }}\right)=-0.592\;\mathrm{CD}-0.164\;\mathrm{UC}$$

This translates to:

$$\hat{\pi }\left(\mathrm{CD},\mathrm{UC}\right)=\frac{\exp\left(-0.592\;\mathrm{CD}-0.164\;\mathrm{UC}\right)}{1+\exp\left(-0.592\;\mathrm{CD}-0.164\;\mathrm{UC}\right)}$$

***Therefore in comparison to the control subjects, CD patients are found have an average 14.4% less (p < .001) Faecalibacterium*** as the following simple calculation shows:

$$\hat{\pi }\left(\mathrm{CD}=1,\mathrm{UC}=0\right)-\hat{\pi }\left(\mathrm{CD}=0,\mathrm{UC}=0\right)=-0.144$$

***Similarly, UC patients are found to have 4.1 % less Faecalibacterium than the control subjects (p = 0.048)*** because $\hat{\pi }\left(\mathrm{CD}=0,\mathrm{UC}=1\right)-\hat{\pi }\left(\mathrm{CD}=0,\mathrm{UC}=0\right)=-0.041$.

**Figure 6:**
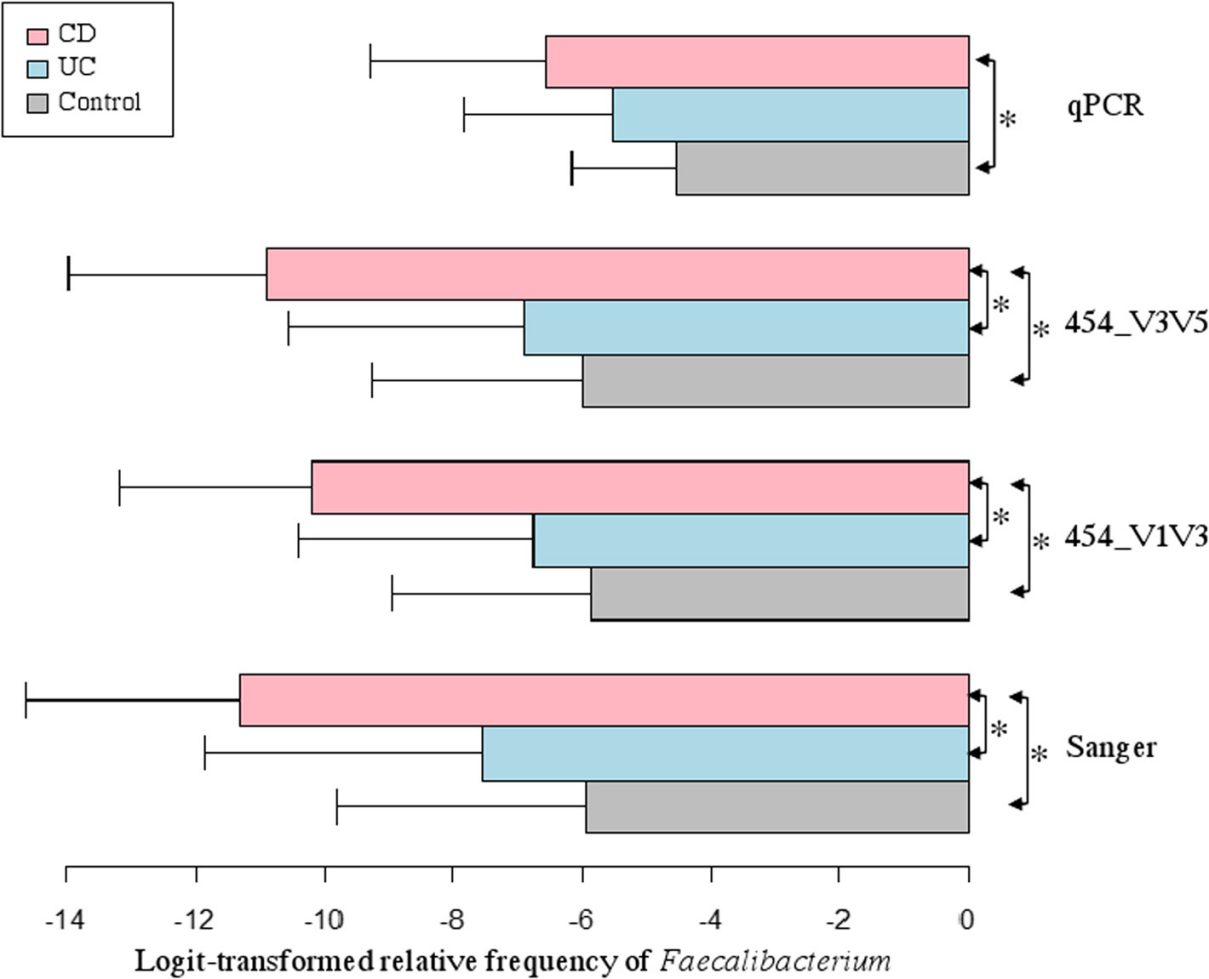
**Comparison of logit-transformed relative frequency of Faecalibacterium among CD, UC and control by four measurements (qPCR, 454_V1V3, 454_V3V5 and Sanger sequencing) respectively.** Mean and standard error are shown on each bar. Pairwise comparisons between UC, CD and control within each measurement platform are performed using Tukey’s studentized range test and significantly different pairs at the familywise error rate of 0.05 are labeled with the asterisk (*) representing significantly different pairs.

The mean differences of the logit-transformed relative frequency of *Faecalibacterium* among CD, UC and control are shown in Figure 6, by Sanger, 454 V1V3, 454 V3V5 and qPCR, respectively. In this case, the trend of decreased relative frequency of *Faecalibacterium* from controls to UC and to CD, appears in agreement among all four measurements. Pairwise comparisons on the relative frequency of *Faecalibacterium* between UC, CD and control within each measurement platform using Tukey’s studentized range test revealed significant difference between CD and controls, while the difference between UC and controls remains insignificant, for all four measurements – consistent to the latent variable SEM results shown in Figure 5(B).

## Conclusions

In this work, we introduced the latent variable SEM as a versatile and effective analytical tool for measurement platform comparison and combination. While traditional SEM relied on the normality assumption for its parametric based inference, thanks to contemporary nonparametric techniques such as the bootstrap resampling method [22,24] and the rapid advancement of modern computers, one can readily perform non-parametric analysis of latent variable SEM when the data are not normal as we have shown in the analysis of a microbiome study of the human inflammatory bowel diseases.

In the study of the gastrointestinal microbiome, we demonstrated that latent variable SEM can provide a robust means of integrating datasets derived from different experimental platforms. Moreover, it can gauge effectively the relative merits of different measurement platforms, in this example, Sanger sequencing, 454 pyrosequencing with two different target regions/windows, and qPCR. Joint panel studies [4] have shown that different 454 pyrosequencing windows may be optimal for different bacterial taxa. Their observations have been confirmed by our own analysis using the latent variable SEM measurement models (Table 4) based on the given IBD study – where the 454_V3V5 window are shown to be a better measurement platform for *Proteobacteria*, *Actinobacteria*, *Bacteroidetes* and *Firmicutes/Bacilli* in addition to the *Faecalibacterium*, while the 454_V1V3 window is found more reliable for *Firmicutes/Clostridia/ Clostridiales/LachnoIV*.

The joint study panel has also recommended sequencing microbiome with two 454 pyrosequencing windows such as V1V3 and V3V5 – which we can readily combine using the latent variable SEM for a unified joint analysis. Nevertheless, more works need to be done for a thorough treatment of the platform comparison problem. For example, we have yet to examine the rare taxa issue. Given that data from rare taxa will feature near zero counts and artificially low or suspiciously high variances, a robust version of the current latent SEM method needs to be developed for the occasion. We definitely expect to submit a follow-up paper on this issue.

To our knowledge, this is the first application of latent variable SEM to the study of human microbiome, and for modern sequencing platform comparison and combination. Since human gastrointestinal microbial communities are typically complex and difficult to study *in situ*, multiple experimental/measurement modalities are required to provide a deep description of the dynamic microbe-microbe and microbe-host interactions in the gut. Given the rapid evolution of modern sequencing technologies, with the debut Sanger sequencing quickly followed by the higher throughput ‘next generation sequencing’ (a.k.a. pyrosequencing) with shorter sequence reads, and with a variety of third and fourth generations sequencing technologies already on the horizon, the platform comparison and combination task is becoming increasingly critical.

## Competing interests

The authors declare that they have no competing interests.

## Authors’ contribution

WZ, KB and XW proposed the statistical methodology. XW carried out data analyses and drafted manuscript. DF and EL provided data interpretation; EL and AG provided experimental data. WZ, KB, DF, EL and AG provided critical revision and suggestive comments of manuscript. All authors read and approved the final manuscript.

## Supplementary Material

Additional file 1: Table S1 Pearson correlations and Text S1 Reliability in the measurement model. Click here for file
