# Supplementary material for: Comparative analysis of microbiome measurement platforms using latent variable structural equation modeling
Source: BMC Bioinformatics. 2013 Mar 5;14:79. doi: 10.1186/1471-2105-14-79 (PMC3608994; doi:10.1186/1471-2105-14-79)
Supplement: Additional file 1: Table S1 — Pearson correlations and Text S1 Reliability in the measurement model. [file 1471-2105-14-79-S1.doc]

**Comparative Analysis of Microbiome Measurement Platforms Using Latent Variable Structural Equation Modeling**

Xiao Wu^1^, Kathryn Berkow^1^, Daniel N. Frank^2^, Ellen Li^3,4^, Ajay S. Gulati^5^, Wei Zhu^1^

^1^Department of Applied Mathematics and Statistics, Stony Brook University, Stony Brook, NY; ^2^Division of Infectious Diseases, University of Colorado Anschutz Medical Campus, Aurora, CO; ^3^Department of Medicine, Stony Brook University, Stony Brook, NY; ^4^Department of Medicine, Washington University, St. Louis, MO. ^5^Department of Pediatrics, University of North Carolina, Chapel Hill, NC.

SUPPLEMENTARY MATERIALS

Table S1. Pearson correlations between three different measurements (Sanger, 454_V1V3 and 454_V3V5) of various bacterial taxa. Bacterial relative frequencies were logit transformed (N = 142).

*(A)* Proteobacteria

| Pearson Correlation | Sanger | 454_V1V3  (*p* value) | 454_V3V5  (*p* value) |
| --- | --- | --- | --- |
| Sanger | 1 | 0.649  (<.001) | 0.800  (<.001) |
| 454_V1V3 |  | 1 | 0.790  (<.001) |
| 454_V3V5 |  |  | 1 |

*(B)* Firmicutes/Clostridia/Clostridiales/LachnoIV

| Pearson Correlation | Sanger | 454_V1V3  (*p* value) | 454_V3V5  (*p* value) |
| --- | --- | --- | --- |
| Sanger | 1 | 0.795  (<.001) | 0.737  (<.001) |
| 454_V1V3 |  | 1 | 0.855  (<.001) |
| 454_V3V5 |  |  | 1 |

(C) *Actinobacteria*

| Pearson Correlation | Sanger | 454_V1V3  (*p* value) | 454_V3V5  (*p* value) |
| --- | --- | --- | --- |
| Sanger | 1 | 0.705  (<.001) | 0. 717  (<.001) |
| 454_V1V3 |  | 1 | 0. 868  (<.001) |
| 454_V3V5 |  |  | 1 |

(D) *Bacteroidetes*

| Pearson Correlation | Sanger | 454_V1V3  (*p* value) | 454_V3V5  (*p* value) |
| --- | --- | --- | --- |
| Sanger | 1 | 0.753  (<.001) | 0.819  (<.001) |
| 454_V1V3 |  | 1 | 0.901  (<.001) |
| 454_V3V5 |  |  | 1 |

*(E)* Firmicutes/Bacilli

| Pearson Correlation | Sanger | 454_V1V3  (*p* value) | 454_V3V5  (*p* value) |
| --- | --- | --- | --- |
| Sanger | 1 | 0.816  (<.001) | 0.818  (<.001) |
| 454_V1V3 |  | 1 | 0.956  (<.001) |
| 454_V3V5 |  |  | 1 |

Text S1. Reliability in the measurement model of latent variable SEM

For the latent SEM model illustrated in Figure 1(A), we have:

$$\begin{matrix} R_{y_{i}}^{2}=\rho_{y_{i},\xi}^{2}\begin{matrix} & & (\text{squared correlation between observed and latent variable}) & \end{matrix} \\ =\frac{\mathrm{cov}^{2}(y_{i},\xi)}{Var(y_{i})Var(\xi)}=\frac{\mathrm{cov}^{2}(\lambda_{i}\xi+\varepsilon_{i},\xi)}{Var(\lambda_{i}\xi+\varepsilon_{i})Var(\xi)}\begin{matrix} & & (∵y_{i}=\lambda_{i}\xi+\varepsilon_{i} \end{matrix}) \\ =\frac{[\lambda_{i}Var(\xi)+\mathrm{cov}(\varepsilon_{i},\xi)]^{2}}{[\lambda_{i}^{2}Var(\xi)+\mathrm{cov}(\lambda_{i}\xi,\varepsilon_{i})+Var(\varepsilon_{i})]Var(\xi)} \\ =\frac{[\lambda_{i}Var(\xi)]^{2}}{[\lambda_{i}^{2}Var(\xi)+Var(\varepsilon_{i})]Var(\xi)}\begin{matrix} & & & \begin{matrix} & & (∵\varepsilon_{i}\perp\xi\end{matrix} \end{matrix},\mathrm{cov}(\varepsilon_{i},\xi)=0) \\ =\frac{\lambda_{i}^{2}Var(\xi)}{\lambda_{i}^{2}Var(\xi)+Var(\varepsilon_{i})}=\frac{\lambda_{i}^{2}Var(\xi)}{Var(y_{i})}=\frac{Var(\lambda_{i}\xi)}{Var(y_{i})}=1-\frac{Var(\varepsilon_{i})}{Var(y_{i})} \end{matrix}$$

The last term in the equation can be interpreted as the proportion of variance in the measure Y_i_ that is explained by the latent variable$\xi$.
